# Aging-associated inflammation and fibrosis in arachnoid membrane

**DOI:** 10.1186/s12883-021-02202-y

**Published:** 2021-04-21

**Authors:** Hime Suzuki, Takeshi Mikami, Naotoshi Iwahara, Yukinori Akiyama, Masahiko Wanibuchi, Katsuya Komatsu, Rintaro Yokoyama, Tsukasa Hirano, Ryusuke Hosoda, Yoshiyuki Horio, Atsushi Kuno, Nobuhiro Mikuni

**Affiliations:** 1 Department of Neurosurgery, Sapporo Medical University, Sapporo, Hokkaido Japan; 2 Department of Pharmacology, Sapporo Medical University, Sapporo, Hokkaido Japan; 3 Department of Neurosurgery, Osaka Medical and Pharmaceutical University, Takatsuki, Osaka Japan

**Keywords:** Arachnoid membrane, Inflammation, Fibrosis, Aging, Cytokine

## Abstract

**Background:**

The physiological and pathological significance of the arachnoid membrane (AM) is still unknown. In this study, we investigated various characteristics of the AM, focusing on the influence of inflammation and fibrosis.

**Methods:**

Small pieces of AM sample were obtained during neurosurgical procedures from 74 cases. The clinical and pathological characteristics of the hyperplastic AM group (≥ 50 μm) and the non-hyperplastic AM group (< 50 μm) were compared. Then, potential correlations between AM thickness and clinical characteristics were analyzed. Moreover, VEGFα, TGFβ, and TGFα levels were quantitated by real time PCR. Then, the potential correlations between AM thickness and these inflammatory or anti-inflammatory markers, and the influence of the original disease were calculated.

**Results:**

The median age of the patients in hyperplastic AM group was significantly older than that of the non-hyperplastic AM group. Moreover, the number of fibroblasts, CD68^+^ cells, CD86^+^ cells, and CD206^+^ cells in the hyperplastic AM group was significantly higher than that in the non-hyperplastic AM group. The AM thickness was significantly correlated to age and number of fibroblasts, CD68^+^ cells, CD86^+^ cells, and CD206^+^ cells. The thickness of the AM was significantly correlated to the messenger RNA expression levels of VEGFα (ρ = 0.337), and the VEGFα expression levels were significantly correlated with TGFβ and TNFα.

**Conclusions:**

The AM hyperplasia was influenced by aging and could be a result of inflammation and fibrosis through cytokine secretion from the inflammatory cells and fibroblasts in the AM.

## Background

The arachnoid membrane (AM) is a tissue named “choroid meninx” by Herophilus in 3 B.C. [1]. Presently, the AM is considered to exist between the dura matter and pia matter and forms a subarachnoid cavity that plays a role in cerebrospinal fluid circulation. From the viewpoint of pathology, the AM comprises two laminar structures: the outer layer and inner layer [1, 2]. The outer layer is the arachnoid barrier cell layer constructed with tight junctions, whereas the inner layer is an arachnoid reticular cell layer loosely constructed with denser cytoplasm, intermediate filaments, and mitochondria [2, 3]. These delicate structures constitute the space of cerebrospinal fluid circulation to exclude unnecessary and harmful materials and to act as cushioning material to protect brain tissue [1, 4]. The AM is relatively hyperplastic in the posterior fossa or around the sylvian fissure [1]. However, there are few reports on the pathological and physiological significance of AM characteristics, and the role of the AM is still not elucidated at present.

Recently, low-level and chronic systematic inflammation has been found to influence the onset and recurrence of various lifestyle diseases [5–7]. This chronic inflammation is mainly caused by macrophages and lymphocytes through various cytokines and their polymorphisms including TNFα, IL-6, TGFβ, and VEGF [8–10], and affects the incidence of stroke or cardiovascular events through atherosclerosis [11, 12]. In addition, chronic inflammation induces fibrosis as shown in idiopathic interstitial pneumonias, constrictive pericarditis, and hepatic cirrhosis [13–17]. Such fibrosis can cause organ failure or organopathy in the final stage. The AM is an avascular tissue. Therefore, it is assumed that inflammation is hardly caused in the AM. However, from the neurosurgical perspective, during intracranial microscopic operation, varying opacity of the AM can be recognized after opening the dura, and we hypothesized that the degree of transparency of the AM might be associated with chronic inflammation and fibrosis. In this study, we investigated the various characteristics of the AM focusing on the influence of chronic inflammation and fibrosis.

## Methods

### Description of patient population

This study was approved by the Ethics Committee of Sapporo Medical University Hospital (no. 292–128), the Ethics Committee of Sapporo City General Hospital (H30–057-449), the Ethics Committee of Shinsapporo Neurosurgical Hospital (2018–1), the Ethics Committee of Oji General Hospital (OGH2018–16) and the Ethics Committee of Hakodate Shintoshi Hospital (2018001), and was performed in accordance with the ethical standards of the 1964 Declaration of Helsinki and its later amendments. This study was a prospective and observational study. Five institutes including our institute with sufficient surgical experience participated in the study. All patients provided informed consent before participating. From August 2017 to August 2019, the AM was obtained during neurosurgical procedure. The study included all cases in which informed consent was provided within a specified period without limiting disease specificity. In the patients with vascular disease, only the AM was obtained, whereas cerebral cortex samples were obtained along with AM in patients with brain tumor, cerebral infarction, and severe trauma. AM sampling was performed above the middle frontal gyrus, and was avoided around the Sylvian fissure, superior sagittal sinus, posterior fossa, and basal cistern because these parts are physiologically hyperplastic. The AM above the cortical artery (prefrontal or precentral artery) was dissected under a microscope, and small pieces (10–30 mm^2^) of the sample were obtained. Using the obtained samples, we assessed the following: (1) pathological characteristics of the AM, (2) messenger RNA expression of the cytokines.

### Preparation of the AM and pathological assessment

For measuring the thickness of the AM in each sample, the specimens needed to be flattened and then embedded in 6.5% Sabouraud agar (Nissui Pharmaceutical Co., Ltd., Tokyo, Japan) as a 1.0 cm square cube sample. For flattening the sample, 4 points of the corners of the AM were sewn on a silicon sheet (5MS01, Kono Seisakusho Co Ltd., Ichikawa, Japan) under a microscope using 10–0 nylon. After sewing onto the silicon sheet, the sample was infiltrated in formalin-fixation liquid for 5 days, and then embedded in 6.5% Sabouraud agar. Finally, the Sabouraud agar-embedded sample was set in paraffin. The paraffin-embedded specimens were sectioned to a thickness of 3 μm.

Infiltration of inflammatory cells and fibrosis was assessed by H-E and immunohistochemical staining. CD68s (macrophage markers), CD86s (type I [M1] macrophage markers), and CD206s (type II [M2] macrophage markers) were used to assess inflammation. Antibodies against the following antigens were used for assessment: CD68 (M0876 Dako clone PG-M1; Agilent Technologies, Santa Clara, CA, USA, 1:100); rabbit anti-CD86 (ab53004, Abcam plc, Cambridge, UK, 1:500); rabbit anti-mannose receptor antibody (anti-CD206; ab64693, Abcam plc, 1:200). In each sample with H-E stain, the thickness of AM was measured in 3 places and the mean value was calculated (HPF, × 400). The number of fibroblasts was then counted in 3 areas of each glass slide (HPF, × 400), and the mean value was calculated. In addition, the local existence of fibroblasts was assessed. Next, the number of CD68^+^ cells, CD86^+^, and CD206^+^ cells were counted in 3 areas using immunohistochemical staining, and the mean value was similarly calculated.

### Stratification of clinical data

Patient clinical data were stratified based on the following 11 clinical variables: (1) age; (2) sex; (3) disease; (4) smoke; (5) systolic blood pressure; (6) total cholesterol; (7) high density lipoprotein (HDL) cholesterol; (8) low-density lipoprotein (LDL) cholesterol; (9) glucose metabolism disorders; (10) a family history of coronary artery disease; and (11) degree of atherosclerosis. The degree of atherosclerosis was determined using the ASCVD score and Suita score [18, 19]. The ASCVD score was advocated in the American College of cardiology and the American Heart Association guideline to evaluate prediction of the 10-year risk of development of ASCVD events. Suita score was advocated by the Japan Atherosclerosis Society guidelines for prevention of atherosclerotic cardiovascular diseases. The clinical and pathological factors were compared between the hyperplastic AM group (≥ 50 μm) and non-hyperplastic AM group (< 50 μm).

### mRNA expression analysis using real-time qRT-PCR

Only AM was used to assess mRNA expression. Samples with adhered blood components or brain tissue were excluded in this analysis. First, RNA extraction was performed using the RNeasy Fibrous Tissue Mini Kit (QIAGEN, Hilden, Germany) according to the manufacturer’s instructions. The total RNA concentration and the A260/A280 ratio were measured using a NanoDrop Lite spectrophotometer (Thermo Fisher Scientific Inc., Waltham, MA, USA). Samples in which the A260/A280 ratio was less than 1.30 were excluded. GoScript™ Reverse Transcription Kit (Promega Corporation, WI, USA) was used to reverse-transcribe 500 ng of total RNA. PCR was performed in a 10 μL volume with 2 μL of cDNA diluted 1:5. TaqMan Universal Master Mix II with UNG and TaqMan Gene Expression Assays for glyceraldehyde 3-phosphate dehydrogenase (GAPDH; Hs02786624_g1), vascular endothelial growth factor A (VEGFα; Hs00998133_m1), transforming growth factor beta 1 (TGFβ; Hs00998133_m1), and tumor necrosis factor (TNFα; HS00174128) were purchased from Thermo Fisher Scientific Inc. qRT-PCR was performed in duplicate on the Applied Biosystems StepOnePlus™ (Thermo Fisher Scientific Inc., Tokyo, Japan). The thermal cycling protocol consisted of 50 °C for 2 min and 95 °C for 10 min, followed by 40 cycles of 95 °C for 15 s and 60 °C for 1 min. The calibration curve of a reference sample with a known level of mRNA expression was used to calculate the expression level of each mRNA. Based on relative quantification, the expression of each mRNA was revised as the ratio to the reference mRNA expression (target mRNA expression/GAPDH expression). Using the mRNA expression data, correlations between the thickness of the AM and mRNA expression levels of TNFα, TGFβ, and VEGFα were investigated.

### Statistical analysis

Data are expressed as median (interquartile range). The Mann-Whitney U test was used for comparison between the hyperplastic AM and non-hyperplastic AM groups. In addition, the male-to-female ratio and presence of clinical characteristics (disease, smoking, glucose metabolism disorders, and a family history of CAD) were compared between the hyperplastic AM and non-hyperplastic AM groups using Fisher’s exact probability test. Simple regression analysis was used to compare the thickness of the AM and the potential factors or mRNA expression levels of cytokines. Spearman’s correlation coefficient was used to calculate the correlation coefficients. A correlation coefficient (ρ) larger than 0.7 indicated a strong correlation, a ρ value between 0.5 and 0.7 indicated moderate correlation, and a ρ < 0.5 indicated weak correlation. All statistical analyses were performed using the SPSS software package (version 24.0, IBM Corp., NY, USA), and statistical significance was set at *p* < 0.05.

## Results

### Patient data

This study included 98 cases operated at our Hospital and an affiliated hospital from September 2017 to April 2019. Twenty-three cases could not be measured because the specimens were over-minute, or due to thermal denaturation during the preparation process. One case was excluded because the AM could not be separated from the meningioma. Consequently, 74 cases could be used to measure the thickness of the AM. Of these 74 cases, 17 cases had partially missing clinical data. The messenger RNA (mRNA) expression levels were measured completely in 57 patients. Of the 74 patients, 44 cases suffered from cerebrovascular disease, 21 cases had brain tumor, and 9 cases were of other diseases. Of the 44 cases of cerebrovascular disease, 14 cases were unruptured aneurysms, 13 cases were of moyamoya disease, 10 cases were of atherosclerotic steno-occlusive disease, 4 cases were of cerebral hemorrhage, and 3 cases were of cerebral arteriovenous malformation. Of the 21 cases of brain tumor, 7 cases were high grade glioma, 5 cases were meningioma, 3 cases were malignant lymphoma, 2 cases were low grade glioma, 1 case was ependymoma, 1 case was craniopharyngioma, 1 case was pituitary adenoma, and 1 case was metastatic carcinoma. The median (interquartile range) age of the patients at the time of surgery was 54.0 years (39.0–68.0). Thirty-one patients were male and 43 were female. The AM was collected from the frontal lobe in 29 cases, the temporal lobe in 35 cases, parietal lobe in 3 cases, occipital lobe in 4 cases, and from the cerebellum in 3 cases. The median Atherosclerotic Cardiovascular Disease (ASCVD) score and Suita score was 6.0 (2.1–24.3) % and 41.0 (23.0–51.3), respectively.

### Pathological characteristics of the AM

**Fig. 1:**
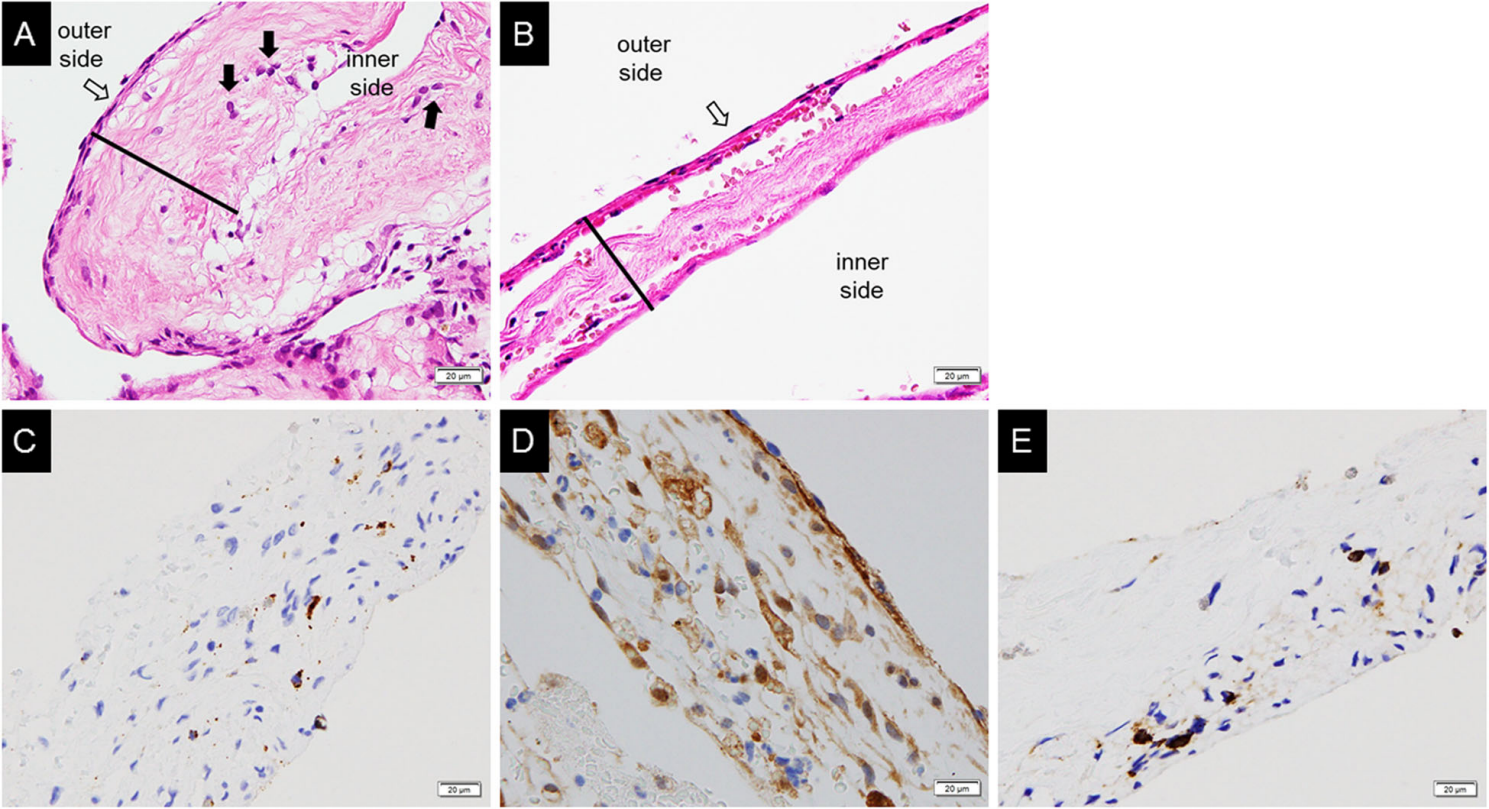
Pathological findings of the AM (× 400) in a representative case of hyperplastic AM (**a**), and non-hyperplastic AM (**b**). The arachnoid membrane includes a barrier cell layer beside the dura matter (white arrow). The fibroblast cells increased and localized beside the inner layer (black arrow). The membrane thickness was measured as the black bar. Immunostaining of the AM (× 400); Macrophages were stained with CD68 (**c**), CD86 (**d**), and/or CD206 (**e**). The number of positive cells for CD68, CD86, and CD206 staining were counted (black arrow). AM: arachnoid membrane

H-E staining showed fibroblasts, fibers, and the barrier cell layer in the AM. Fibroblasts tend to exist at the inward pial side, and the fibroblast decreased at the outward dural side being replaced by fibers (Fig. 1a, b). In the 16 specimens including brain tissue, hyperplasia of the AM was observed around the arteries in 6 cases. CD68^+^, CD86^+^, CD206^+^ staining showed the infiltration of macrophages, M1 macrophages and M2 macrophages respectively, in the AM (Fig. 1c, d, e). CD68^+^ cells and CD206^+^ cells tended to be accumulated at inward pial side. The median thickness of AM was 49.62 (34.05–69.13) μm. The median number of fibroblasts counted by Hematoxylin-Eosin (H-E) stain was 88.48 (41.14–125.97) × 10^− 3^/μm^2^. A total of 46 cases were stained positively for CD68, and the median number of CD68^+^ cells was 2.85 (0.00–12.56) × 10^− 3^/μm^2^. Of these 46 cases stained positively for CD68, 13 cases were stained positively for CD86 as well, and the median number of CD86^+^ cells was 0.00 (0.00–0.00) × 10^− 3^/μm^2^. Additionally, 32 cases were stained positively for CD206, and the median number of CD206^+^ cell was 0.00 (0.00–10.24) × 10^− 3^/μm^2^.

**Fig. 2:**
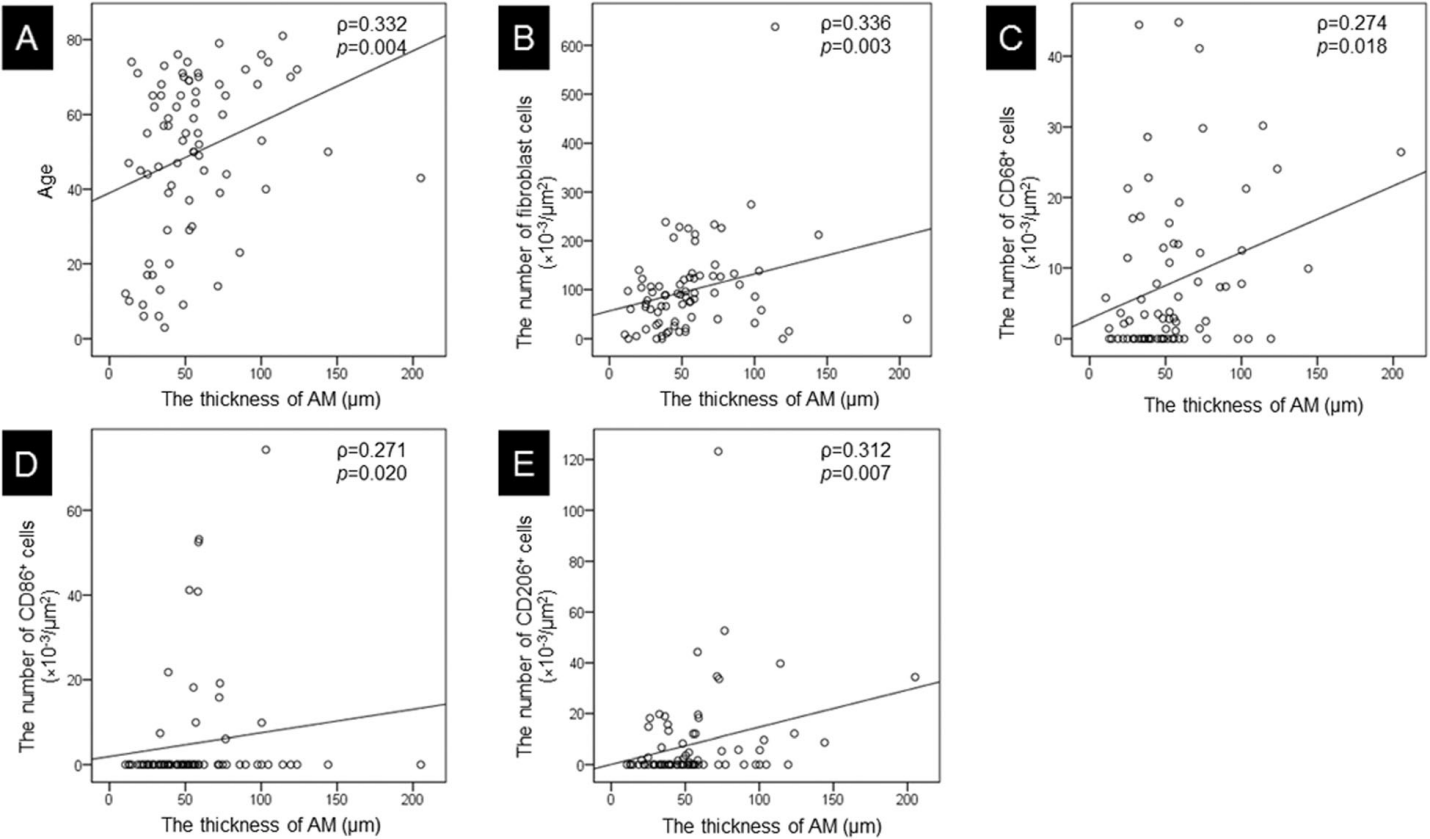
Correlation between the thickness of the AM and age (**a**), fibroblasts (**b**), CD68^+^ cells (**c**), CD86^+^ cells (**d**), and CD206^+^ cells (**e**) cells. The correlation between these were significant (ρ = 0.332, *p* = 0.004; ρ = 0.336, *p* = 0.003; ρ = 0.274, *p* = 0.018; ρ = 0.271, *p* = 0.020; ρ = 0.312, *p* = 0.007), respectively. AM: arachnoid membrane

Table 1 shows the clinical and pathological characteristics associated with the AM thickness. We divided patients into two groups; hyperplastic AM group (≥ 50 μm) and non-hyperplastic AM group (< 50 μm). The hyperplastic AM group and the non-hyperplastic AM group included 37 cases each. The median age of the patient was 59.0 years (45.0–70.0) in the hyperplastic AM group, and 47.0 years (17.0–65.0) in the non-hyperplastic AM group indicating that the older patients exhibited hyperplastic AM (*p* = 0.020). The AM thickness was significantly correlated to age (ρ = 0.332, *p* = 0.004) (Fig. 2a). Patients’ sex did not affect the hyperplasia of the AM (*p* = 0.814). With regards to other clinical factors including disease, smoke, systolic blood pressure, total cholesterol, HDL cholesterol, LDL cholesterol, glucose metabolism disorders, and familial history of coronary artery disease, there was no significant difference between hyperplastic AM group and non-hyperplastic AM group. The median ASCVD score in the hyperplastic AM group [11.1 (2.2–27.2) %] was higher than that in the non-hyperplastic AM group [4.3 (1.2–16.4) %], though it was not significant (*p* = 0.176). The median Suita score in the hyperplastic AM group [45.0 (31.00–54.00)] was higher than that in the non-hyperplastic AM group [40.0 (− 1.50–48.50)] and was also not significant (*p* = 0.153). The median number of fibroblasts in the hyperplastic AM group and non-hyperplastic AM group were 110.16 (69.83–138.35) × 10^− 3^/μm^2^ and 65.84 (19.28–97.01) × 10^− 3^/μm^2^, respectively, with a significant difference between the 2 groups (*p* = 0.004). The AM thickness was significantly correlated to the number of fibroblasts (ρ = 0.336, *p* = 0.003) (Fig. 2b). The median number of CD68^+^ cells in the hyperplastic AM group and non-hyperplastic AM group was 7.28 (1.12–13.46) × 10^− 3^/um^2^ and 0.00 (0.00–5.75) × 10^− 3^/μm^2^, respectively, with a significant difference (*p* = 0.023). The AM thickness was significantly correlated to the number of CD68^+^ cells (ρ = 0.274, *p* = 0.018) (Fig. 2c). The median number of CD86^+^ cells in the hyperplastic AM group and non-hyperplastic AM group was 0.00 (0.00–9.83) × 10^− 3^/μm^2^ and 0.00 (0.00–0.00) × 10^− 3^/μm^2^, respectively, with a significant difference (*p* = 0.005). The AM thickness was significantly correlated to the number of CD86^+^ cells (ρ = 0.271, *p* = 0.020) (Fig. 2d). The median number of CD206^+^ cell in the hyperplastic AM group and non-hyperplastic AM group was 3.63 (0.00–12.11) × 10^− 3^/μm^2^ and 0.00 (0.00–2.34) × 10^− 3^/μm^2^, respectively, with a significant difference (*p* = 0.020). The AM thickness was significantly correlated to the number of CD206^+^ cells (ρ = 0.312, *p* = 0.007) (Fig. 2e). These findings suggested that fibrosis and chronic inflammation might be seen, especially in patients with hyperplastic AM.

**Table 1:** Comparison of the characteristics of patients in the hyperplastic AM group and the non-hyperplastic AM group

Median (interquartile range)	Total	AM thickness		*P* value
		Hyperplastic AM (50 μm≧)	Non-hyperplastic AM (< 50 μm)	
AM thickness (μm)	49.62 (34.05–69.13)	71.42 (55.68–97.60)	33.99 (24.96–39.39)	**< 0.001**
Age	54.00 (39.00–68.00)	59.00 (45.00–70.00)	47.00 (17.00–65.00)	**0.020**
Sex (male/female)	31/42	16/21	15/22	0.814
Disease				
Cerebrovascular disease	44	23	21	0.192
Brain tumor	21	12	9	
Others	9	2	7	
Smoke (yes/no)	34/40	20/17	14/23	0.162
Systolic BP (mmHg)	120.00 (110.00–132.50)	130.00 (110.00–140.00)	120 (110.00–130.00)	0.249
Total Cholesterol (mg/dL)	201.00 (168.50–233.00)	205.00 (176.00–234.50)	187.00 (164.00–228.00)	0.465
HDL (mg/dL)	54.00 (42.50–67.00)	52.50 (42.50–65.00)	58.00 (43.00–70.00)	0.322
LDL (mg/dL)	121.00 (85.00–136.80)	126.00 (85.00–146.00)	107.00 (88.20–130.00)	0.335
Glucose metabolism disorders (yes/no)	15/59	7/30	8/29	0.772
A family history of CAD (yes/no)	1/72	1/36	0/36	0.321
ASCVD score [%]	6.00 (2.10–24.30)	11.10 (2.20–27.20)	4.30 (1.15–16.35)	0.176
Suita score	41.00 (23.00–51.30)	45.00 (31.00–54.00)	40.00 (−1.50–48.50)	0.153
Fibroblasts (×10^−3^/μm^2^)	88.48 (41.14–125.97)	110.16 (69.83–138.35)	65.84 (19.28–97.01)	**0.004**
CD68^+^ cell (×10^−3^/μm^2^)	2.825 (0.00–12.57)	7.28 (1.12–13.46)	0.00 (0.00–5.75)	**0.023**
CD86^+^ cell (×10^−3^/μm^2^)	0.00 (0.00–0.00)	0.00 (0.00–9.83)	0.00 (0.00–0.00)	**0.005**
CD206^+^ cell (×10^−3^/μm^2^)	0.00 (0.00–10.25)	3.63 (0.00–12.11)	0.00 (0.00–2.34)	**0.020**
TGFβ [fold change]	0.50 (0.03–1.76)	0.61 (0.00–2.13)	0.48 (0.18–1.63)	0.445
TNFα [fold change]	48.02 (0.00–1361.71)	114.89 (0.00–1008.17)	32.12 (0.00–1428.72)	0.936
VEGFα [fold change]	0.68 (0.09–2.24)	1.30 (0.29–2.85)	0.23 (0.07–1.60)	**0.043**

*AM* Arachnoid membrane, *CAD* Coronary artery disease, *ASCVD* Atherosclerotic Cardiovascular Disease

### mRNA expression of cytokines

**Fig. 3:**
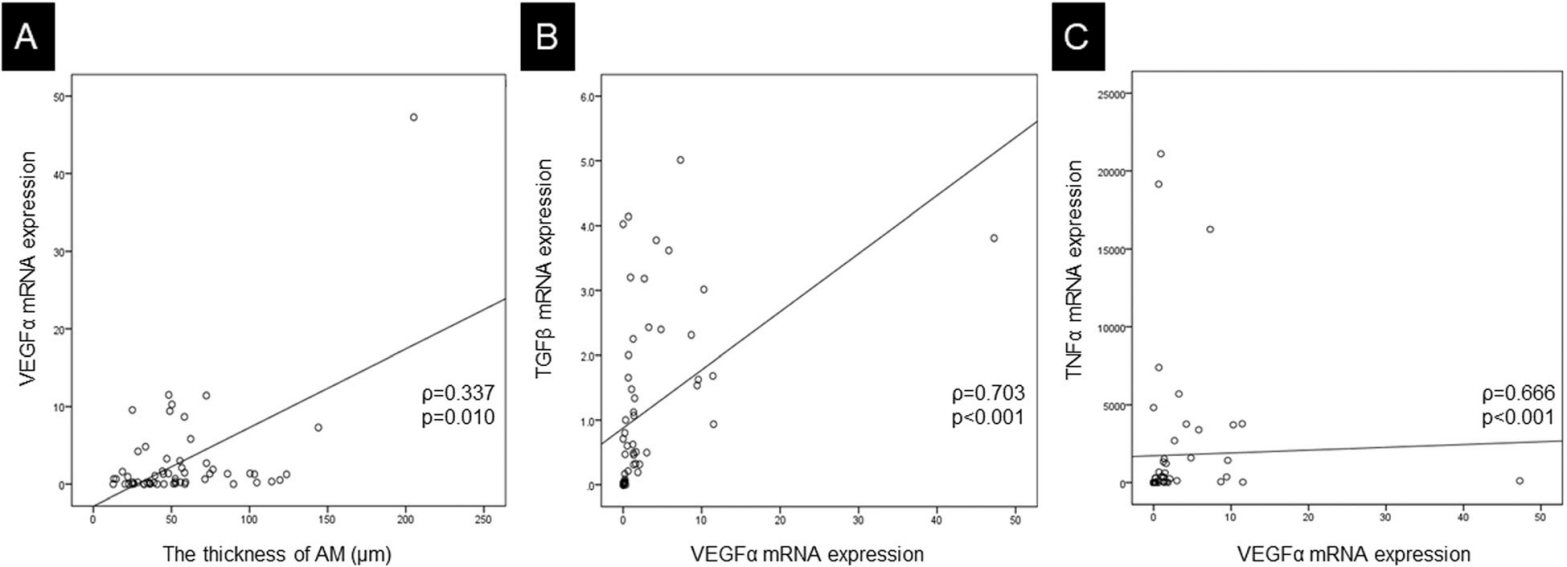
Correlation between the thickness of AM and the expression levels of VEGRα, TGFβ, and TGFα mRNA. The AM thickness was significantly correlated with the mRNA expression of VEGFα (ρ = 0.337, *p* = 0.010) (**a**). VEGFα mRNA expression was significantly correlated to TGFβ mRNA expression (ρ = 0.703, *p* < 0.001) (**b**), and TNFα mRNA expression (ρ = 0.666, *p* < 0.001) (**c**). AM: arachnoid membrane

To investigate whether biological factors have the potential to influence the thickness of AM, we measured the mRNA levels of VEGFα and the thickness of AM. mRNA expression levels were measured in 58 cases, including 28 and 30 cases in the hyperplastic AM and non-hyperplastic AM groups, respectively. First, VEGFα was assessed because VEGFα is a key factor for tissue repair that is released by fibroblasts and inflammatory cells. The median value of VEGFα mRNA expression was 0.68 (0.09–2.24). The median value of VEGFα mRNA expression levels was 1.30 (0.29–2.85) in the hyperplastic AM group, and 0.23 (0.07–1.60) in the non-hyperplastic AM group (*p* = 0.043). VEGFα mRNA expression levels were significantly correlated with the thickness of the AM (ρ = 0.337, *p* = 0.010) (Fig. 3a). These findings indicated that AM hyperplasia is associated with VEGFα expression.

Next, TNFα and TGFβ were compared with the thickness of the AM and VEGFα expression, because these are representative inflammatory mediators that influence VEGFα. Although the median values of TNFα and TGFβ mRNA expression levels were not significantly different between the hyperplastic AM group and the non-hyperplastic AM group (*p* = 0.936 and 0.445 respectively), VEGFα mRNA expression was significantly correlated with TGFβ mRNA expression (ρ = 0.703, *p* < 0.001) (Fig. 3b). In addition, VEGFα mRNA expression was significantly correlated with TNFα mRNA expression (ρ = 0.666, *p* < 0.001) (Fig. 3c). These findings indicate that other inflammatory mediators, including TNFα and TGFβ, are associated with VEGFα and might indirectly influence AM thickness.

## Discussion

In the AM, hyperplasia due to fibroblast proliferation, and infiltration of CD68^+^ cells, CD86^+^ positive cells, and CD206^+^ cells were observed, suggesting the presence of chronic inflammation focusing on macrophages in the AM. Macrophages are a key regulator of chronic inflammation and fibrosis in various disorders such as pericarditis, and hepatic cirrhosis and arteriosclerotic plaque [20–22], and in this study, among all cases, 63.5% involved CD68^+^ cells. In the AM, inflammatory and anti-inflammatory cells should be derived from the cerebrospinal fluid of the subarachnoid space, and/or the cerebral arteries and brain tissue. The macrophages are divided into two phenotypes varying in their physiological roles: M1 and M2 macrophages [23, 24]. The M1 macrophage appears in inflammatory conditions and promotes damage of the tissue organization by producing pro-inflammatory cytokines (IL-1b, TNFα, IL-6, and IL-12) [25]. On the contrary, the M2 type macrophages appear in anti-inflammatory conditions and produces anti-inflammatory cytokines (IL-10, IGF-1, and TGFβ) working in phagocytosis and restoration of tissue organization [25]. The distribution of various macrophage phenotypes is considered to allow prediction of the inflammatory stage. In atherosclerosis, some reports show that the dominance of M1 macrophages results in a vicious cycle of plaque formation [26]. In this study, 17.6% were CD86^+^ cells and 43.2% were CD206^+^ cells, and the M2 marker was superior. The M2 macrophages facilitate healing and repairing of the inflammatory process, show anti-inflammatory action, and promote fibrosis [23]; it is thought that these cells contribute to hyperplasia of the AM. Further, TGFβ and TNFα in the AM were strongly correlated in this study. M1 and M2 macrophages co-exist in various inflammatory states, and dynamic changes in the M1/M2 phenotype of recruited mononuclear phagocytes have been observed in other disease model as well [27–29].

**Fig. 4:**
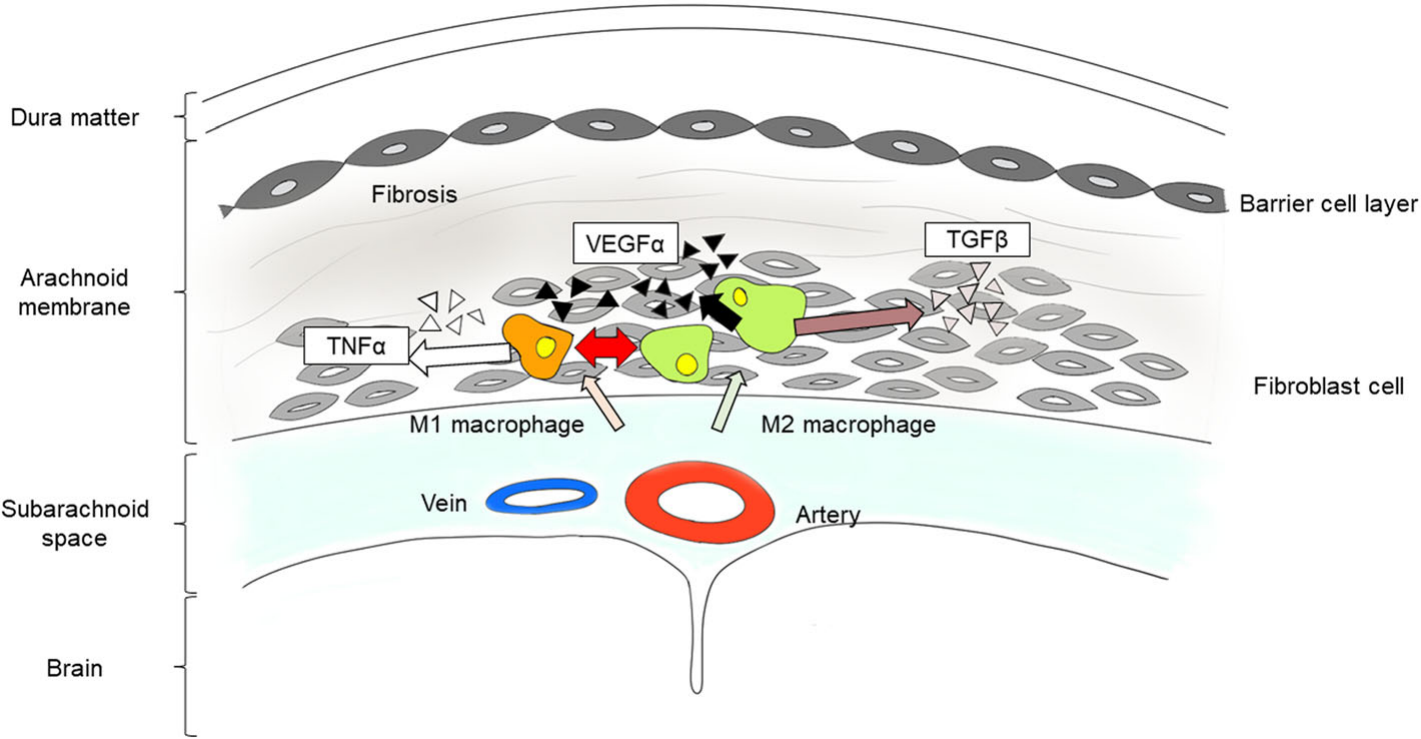
Illustration of the process of AM hyperplasia. Fibroblasts and VEGFα play an important role in AM hyperplasia. Infiltration of macrophages though chronic inflammation tends to distribute inward to the pial side adjacent to the brain surface, and the extracellular matrix including collagen fibers mainly exists at the outward dural side. Macrophage activation accelerates the elevation of TNFα and TGFβ and promotes fibrosis. Consequently, VEGFα levels increase in the AM. The depicted image is our own design. AM: arachnoid membrane

Fibroblast proliferation is observed in the regeneration process of chronic inflammation due to anti-inflammatory cytokines [30]. Our results indicate that the infiltration of chronic inflammatory cells and fibroblasts tends to distribute in the inner layer adjacent to the brain surface, and that extracellular matrix including collagen fibers mainly exists at the dura matter side, in the barrier cell layer. It was considered that the AM hyperplasia originates from the inner side of the AM, and that inflammation begins from the subarachnoid space near the arteries (Fig. 4). TGFβ causes tissue fibrosis due to the proliferation of fibroblasts and production of extracellular matrix [31]. In this study, VEGFα levels correlated with the thickness of the AM. VEGFα is produced by several types of cells including fibroblasts and inflammatory cells [32, 33]. In moyamoya disease, high VEGF expression was correlated with TGFβ [34], and might be associated with abnormal vascular hyperplasia. Although the pathological meaning of excessive AM hyperplasia is still obscure, in other organs including the lung, liver, and kidney, excessive fibrosis leads to defective repair and induces organ failure in the final stages [27, 35]. In the brain, fibrosis of the AM might affect the pulsative circulation of cerebrospinal fluid. Recently, the glymphatic system of the perivascular and subarachnoid space has gained attention and has been shown to be associated with Alzheimer’s disease [36]. In future work, understanding the harmful effect of AM hyperplasia on the glymphatic system or cognitive function will be crucial. From the clinical viewpoint, only aging was associated with the AM thickness in this study. Progressive fibrosis is a hallmark of the aging process and has been implicated in the pathogenesis of diseases of the heart, lungs, liver, kidneys, and bone marrow [37]. Contrary to expectations, there were no correlations between the AM hyperplasia and clinical factors, including atherosclerosis scores. In stroke, chronic cerebral ischemic state, brain tumor, and brain abscess, these pathologies should be a state of microglia or macrophage activation [38, 39], though the influence of each pathology was minimal in the AM. Therefore, it will be necessary to compare the groups with equivalent ages to assess the influence of the disease.

There are some limitations to this study. First, this study included various kinds of diseases, and the degree of the disease was different in each case. For instance, local factors might depend on the stage of the original disease especially in extra-axial tumors. On the other hand, it was considered that the AM could be affected widely in chronic ischemic disease, though the degree of the ischemia was different in each case. This study did not exclude the influences of disease specificity. Secondly, the quantity of specimens was not sufficient for analysis by real-time qRT-PCR. Samples in which the A260/A280 ratio was set less than 1.30 were excluded in this study, though their value was relatively low. Third, there were problems in terms of AM fixation. Because AM is a very thin and delicate tissue, thermal denaturation might have occurred in the Sabouraud agar fixation process. Although, an alternative technique is needed to assess the cerebral environment, this could not be achieved to find pathological significance.

In conclusion, AM hyperplasia was influenced by aging. Pathologically, fibroblasts and M2 macrophages proliferated in the inward pia side especially around the arteries. AM hyperplasia was correlated with VEGFα and might be a result of anti-inflammatory changes and fibrosis. These alterations might be affected by cerebral ischemia, especially in moyamoya disease. Further analysis of the AM in various diseases And more number of cases will provide us with answers to some of these hypothesis.

## Conclusion

In conclusion, AM hyperplasia was influenced by aging. Pathologically, fibroblasts and M2 macrophages proliferated in the inward pia side especially around the arteries. AM hyperplasia was correlated with VEGFα and might be a result of anti-inflammatory changes and fibrosis. Further analysis of the AM in various diseases and more number of cases will provide us with answers to some of these hypothesis.

## Data Availability

The datasets used and/or analyzed during the current study are available from the corresponding author on reasonable request. The data are not publicly available due to privacy or ethical restrictions.

## Abbreviations

AM: Arachnoid membrane
mRNA: Messenger RNA
ASCVD: Atherosclerotic Cardiovascular Disease
H-E: Hematoxylin-Eosin
